# “Righting the wrongs”: addressing human rights and gender equality through research since Cairo

**DOI:** 10.1080/26410397.2019.1676529

**Published:** 2019-11-08

**Authors:** Rajat Khosla, Avni Amin, Pascale Allotey, Carmen Barroso, Asha George, Anita Hardon, Ian Askew

**Affiliations:** a Human Rights Adviser, Human Reproduction Programme (HRP), World Health Organization, Geneva, Switzerland; b Scientist, Human Reproduction Programme (HRP), World Health Organization, Geneva, Switzerland; c Director, United Nations University – International Institute for Global Health, Kuala Lumpur, Malaysia; d Interim Executive Director, Global Doctors for Choice, Portland, OR, USA; e SARChI Professor Health Systems, Complexity and Social Change, School of Public Health, University of Western Cape, Cape Town, South Africa; f Professor, Faculty of Social and Behavioral Sciences, University of Amsterdam, Amsterdam, Netherlands; g Director, Human Reproduction Programme (HRP), World Health Organization, Geneva, Switzerland

The Programme of Action (PoA) adopted in Cairo at the International Conference on Population and Development (ICPD) in 1994 called for strengthening of the evidence base to guide policies and programming to deliver on commitments made. The POA emphasised that research should be guided by women’s needs and preferences, based on objectives of advancing gender equality and equity and the empowerment of women, and be carried out in accordance with internationally accepted legal, ethical, medical and scientific research standards.

The following years saw researchers and funding bodies revising their focus to meet the need for evidence that could inform implementation of the PoA. Notable progress includes establishment of dedicated research programmes and consortia on, e.g. violence against women (VAW) (DFID supported); female genital mutilation (Population Council led) and sexual behaviours (London School of Hygiene and Tropical Medicine led). Changes in the way research funding and programming were managed occurred: for instance, within the UN co-sponsored Special Programme of Research on Human Reproduction (HRP), a Gender and Rights Advisory Panel was established in 1996 to guide sexual and reproductive health (SRH) research and inform development of WHO’s normative guidance.^1^ An inclusive, complex understanding of gender equality and human rights evolved, although this identified further future needs for clarity and knowledge. Virtually all research donors now require gender equality and rights be explicitly addressed in applications for funding. We examine how SRH research has evolved in addressing gender equality and human rights and propose future directions, using examples from the work of HRP to illustrate key arguments.

## What difference has research made in addressing gender equality and human rights?

The 2005 WHO Commission on Social Determinants of Health (CSDH),^2^ and more recently the Lancet Series on gender equality, gender norms and health^3^, explain how gender inequality, among other social determinants, influences health risks and vulnerabilities as well as girls' and women's access to services and the gender-specific consequences of ill health. The Commission reinforced the concept of intersectionality, wherein gender interacts with other axes of discrimination, such as race, age, and income, to create unequal power dynamics and hierarchies.

The body of evidence drawn from twenty-five years of research that explicitly addresses gender equality and human rights highlights the centrality of unequal power and male privilege in influencing women’s and girls’ experiences of health and limiting their access to services.^2,3^ Social, economic, cultural and political factors, alongside unequal power relationships between couples, within families and households and in communities, together contribute to maintaining gender inequality resulting in women and girls being disproportionately affected by adverse consequences for SRH.^4^

Research on the manifestations of gender inequality and human rights violations and successful interventions that promote gender equality and human rights on SRH policies, programmes and outcomes, has been instrumental in advancing knowledge and building the evidence that informs normative guidance, including WHO’s guideline development processes, and policy development and programming by countries. The *Kesho Bora* study, for instance, assessed Prevention of Mother-to-Child Transmission (PMTCT) interventions for breastfeeding women in Kenya.^5^ The study was the first in PMTCT to include perspectives of women living with HIV, focusing on expressed needs for treatment and care in the evaluation. Women’s experiences of coercion and violence were documented. The study promoted women’s right to care, influencing WHO guidelines on antiretrovirals and prevention of mother-to-child transmission of HIV, providing hope for mothers with HIV infection who cannot safely feed their babies with infant formula.

Research on measuring the prevalence of VAW a key manifestation of gender inequality and human rights violation, has established standard measures for ethically and safely gathering data, and contributed to a wider understanding of the public health burden on women’s well-being and SRH.^6^ Research on VAW demonstrated that the process of conducting research needs to be women-centred, sensitive to their trauma and address their safety, while also measuring the health burden. Data on VAW is now available for over 100 countries, forming the basis for tracking changes in prevalence and impact of interventions and programmes, including on SRH outcomes.^7^

## Where are we now?

Research that explicitly addresses gender inequalities and human rights can and has made significant differences to SRH policies and programmes since Cairo. Women's rights to access, autonomy, safety, dignity and well-being are recognised as important outcomes. Research has also pointed out that SRHR experiences are affected not only by gender but also by axes of social differentiation – ethnicity, income, sexual orientation etc. Yet gaps in evidence of impact and biases in research processes continue to hamper the systematic integration of gender equality and human rights into SRH research.

To date, research that includes both gender equality and human rights considerations has predominantly been undertaken on HIV/AIDS, maternal health, contraception, and VAW. Although research on sexually transmitted and reproductive tract infections, reproductive cancers, and infertility has also embraced this approach, greater attention to gender equality and rights is needed. A recent review identified two areas for further attention: understanding the pathways by which interventions for gender equality and human rights outcomes can improve SRH outcomes; and addressing intersecting forms of inequalities and discrimination based on gender diversities, social status, sexual orientation, ethnicity and other factors and their impacts on access to SRH services.^8^ Increased coordination between gender and human rights researchers and public health researchers, for example, through research management mechanisms such as HRP’s Gender and Rights Advisory Panel, could further improve the design and implementation of explanatory research studies, intervention evaluations and clinical trials, thereby strengthening the quality, relevance and impact of the evidence base. The extent to which gender equality and human rights are addressed in undergraduate, graduate and in-service training for health researchers is also a limiting factor, highlighting a need for revisions in training curricula.

The use of conventional biomedical, epidemiological and public health research methods without methodologies that offer understanding of gender equality and rights issues limits the type of evidence available. Research methods from the social and political sciences can broaden evidence. Short timeframes, limited geographical coverage and minimal variety of populations sampled compound methodological limitations. Studies addressing gender equality and human rights often do not consider the costs of intervention, their replicability or sustainability at scale.^8^ There is emerging evidence that shows the importance of addressing and measuring these issues.

## Conclusion

Integrating gender equality and human rights perspectives in research on SRH is critical to generate evidence on experiences, needs and preferences of users of SRH services. New challenges - environmental threats, climate change, antimicrobial resistance, epidemiological and demographic shifts - require research to explore intersections with gender equality and rights. Shifts towards social and religious conservatism and growing economic inequalities within and between countries are curtailing human rights and sustaining or increasing gender inequalities.^8^ When these challenges occur in low- and middle-income countries, they are compounded by limited funding for research. Focusing on a few critical actions could make the PoA commitment to improve research a reality in future:

1. Implement and fund research that addresses intersecting forms of discrimination and rights violations that affect the SRH of persons made vulnerable through these intersections;
2. Support research to understand how political will and civil society activism affect SRH policies and programming that seek gender equality and human rights;
3. Establish national research governance mechanisms to ensure independence and accountability of researchers and research funders to those most affected by structural gender inequality and violations of their human rights;
4. Promote use of study designs and analytical approaches that go beyond determining efficacy and efficiency to identify how interventions function in complex real-life contexts and explain the processes by which integration of human rights and gender equality in SRH programmes and policies can improve health outcomes.

## References

[1] Cottingham J. Historical note: how bringing women’s health advocacy groups to WHO helped change the research agenda. Reprod Health Matters. 2015;23(45):12–20. doi: 10.1016/j.rhm.2015.06.007 26278829

[2] IIMB and Karolinska Institutet. Unequal, Unfair, Ineffective and Inefficient. Gender Inequity in Health: Why it exists and how we can change it [Internet]. (2007). https://www.who.int/social_determinants/resources/csdh_media/wgekn_final_report_07.pdf.

[3] Gupta R, Oomman N, Grown C, et al. Gender equality and gender norms: framing the opportunities for health. The Lancet; (2019):393(10190). doi: 10.1016/S0140-6736(19)30651-8 31155276

[4] Hannah FG, Taukobong M. Does addressing gender inequalities and empowering women and girls improve health and development programme outcomes? Health Policy Plann. December 2016;31(10):1492–1514. doi: 10.1093/heapol/czw074 27371549

[5] WHO. Kesho Bora Study Preventing Mother to Child Transmission of HIV during breastfeeding. 2011.

[6] Garcia-Moreno C. WHO. Putting women first: ethical and safety recommendations for research on domestic violence against women. WHO; 2005; 2001.

[7] Amin A, Chandra-Mouli V. Empowering adolescent girls: developing egalitarian gender norms to end violence. Reprod Health. 2014;11(75):11–14. 25335989 10.1186/1742-4755-11-75 PMC4216358

[8] Hartmann M, Khosla R, et al. How are gender equality and human rights interventions included in sexual and reproductive health programmes and policies: a systematic review of existing research foci and gaps. PLoS ONE. 2016;11(12):e0167542. doi: 10.1371/journal.pone.0167542 28002440 PMC5176262

